# Book Reviews

**Published:** 1911-01

**Authors:** 


### BOOK REVIEWS

ANATOMY, DESCRIPTIVE AND APPLIED. By Henry Gray, F. R. S., late lecturer on Anatomy at St. George's Hospital, London. New (18th) edition, thoroughly revised, by Edward Anthony Spitzka, M. D., Professor of Anatomy in the Jefferson Medical College of Philadelphia. Imperial octavo, 1496 pages, with 1208 large and elaborate engravings. Price, with illustrations in colors, cloth, $6.00, net; leather, $7.00, net. Lea & Febiger, Publishers Philadelphia and New York, 1910.

This new edition, the eighteenth, is the most thorough of all revisions, every line having been scrutinized for possible improvement, anything in the natures of an obscurity being clarified, and whole passages rewritten. The Editor, Dr. E. A. Spitzka, is Professor of Anatomy in the Jefferson Medical College of Philadelphia, and one of the world's foremost anatomists. He is also a competent artist, and the drawings from his hand convey his own accurate knowledge directly to the mind of the student. Nothing has been spared to maintain the reputation of the book as being the easiest from which to teach or to learn, and as facilitating to the utmost the acquisition and retention of a sound knowledge of its subject.

---

A TREATISE ON DISEASES OF THE EYE. By John E. Weeks, M. D., Professor of Ophthalmology in the University and Bellevue Hospital Medical College, New York. In one octavo of 944 pages, with 528 illustrations and 25 full-page plates. Cloth, $6.00, net. Lea & Febiger, Philadelphia and New York, 1910.

In this volume an authority of international reputation covers the whole subject of ophthalmology in a manner answering all needs of the student and general practitioner, and also serving the specialist for reference on the latest developments. The comprehensiveness of this single volume is shown by its inclusion of the embryology and anatomy of the eye, and the principles of optics as well as the clinical aspects of the subject, such fundamentals being necessary to a full understanding of the anomalies and diseases of the organ.

---

MODERN TREATMENT; THE MANAGEMENT OF DISEASE WITH MEDICINAL AND NON-MEDICINAL REMEDIES. By Emiennt American and English authors. Edited by Hobart Amory Hare, M. D., Professor of Therapeutics and Materia Medica, Jefferson Medical College, Philadelphia; Physician to the Jefferson Hospital; Author of "A Text-book of Practical Therapeutics," "A Text-book of the Practice of Medicine," etc. In two very handsome octavo volumes, comprising 1800 pages, with numerous engravings and full-page plates. Price per volume, in cloth $6.00, net; half morocco, $7.50, net. Lea & Febiger, Publishers, Philadelphia and New York, 1910.

The decade just closing is notable for the intensity and fruitfulness with which scientific methods of investigation have been brought to bear upon all basic departments of medicine. The very secrets of Nature have been penetrated, and her own modes of combatting disease have been discovered. The time is therefore opportune for a special work on Treatment, written by the highest authorities and so clear and direct that physicians who are not so situated that they can keep abreast of the stream of new knowledge proceeding from the world's laboratory and research workers, may, nevertheless, have the results at command in a shape for immediate application in practice. Such is the object accomplished in this entirely new work. Dr. Hare has known how to plan it and where to find the best man for each subject. The field covered is inclusive of all methods of treating disease.

Page(s) missing

